# Do you have a good all-around view? Evaluation of a decision-making skills diagnostic tool using 360° videos and head-mounted displays in elite youth soccer

**DOI:** 10.3389/fspor.2023.1171262

**Published:** 2023-06-05

**Authors:** Oliver Höner, Damir Dugandzic, Thomas Hauser, Michael Stügelmaier, Nico Willig, Florian Schultz

**Affiliations:** ^1^ Institute of Sports Science, University of Tübingen, Tübingen, Germany; ^2^ DFB Academy, Frankfurt, Germany; ^3^ VfB Stuttgart 1893 AG, Stuttgart, Germany

**Keywords:** talent identification and development, perceptual-cognitive skills, football (soccer), expertise, virtual reality

## Abstract

Elite youth players’ decision-making skills are considered important predictors of adult performance in soccer. The presentation of 360° videos in head-mounted displays offers new potential for the diagnostic of these skills in talent development programs. This study evaluated a new diagnostic tool using soccer-specific 360° videos for assessing decision-making skills in youth academy (YA) players. The evaluation consisted of players' subjective feedback as well as the analysis of diagnostic and prognostic validity. It was hypothesized that high-level YA players achieve better diagnostic results than regional-level players, and U19 outperform U17 players. Moreover, YA players' diagnostic results should be positively associated with future adult performance level. During the 2018/19 season, *N* = 48 youth players participated in the diagnostic procedures (split-half reliability *r *= .78). Participants were shown 54 videos which terminated when the central midfielder received a teammate's pass. Participants were then asked how to best continue playing. The subjective evaluation explored YA players' experiences with the diagnostic tool via quantitative ratings (e.g., “How exciting was the task?”, “How involved did you feel in the game situation?”) and additional interviews. Diagnostic validity was examined in a balanced cross-sectional 2 × 2-design (performance level x age group) and prognostic validity in a 3-year prospective design. Sensitivity and case-by-case analyses completed the evaluation. The YA players provided positive quantitative ratings regarding their experienced immersion into the environment. Players’ qualitative feedback indicated general acceptance of the diagnostic tool as well as it offered recommendations for improvements. Confirming the diagnostic validity, ANOVA revealed significant main effects for performance level (*p *< .001, *η*^2^ = .29) and age group (*p *< .01, *η*^2^ = .14). Contributing to the prognostic validity, the diagnostic results discriminated between YA players achieving a higher and a lower adult performance level (“League 1–4” vs. “League 5 or below”) in adulthood (*p *< .05; *d *= 0.80). A ROC curve and the AUC showed that the correct assignment to the adult performance levels is possible with a 71% probability. YA players with a high decision-making accuracy had a six times higher chance of playing in “League 1–4”. The results demonstrated empirical evidence for the new diagnostic tool in terms of YA players' acceptance and validity coefficients exceeding effect sizes of former studies. The technology provides opportunities to test soccer-specific situations demanding an all-around view that were not testable in former experimental settings. Further technological advancements will enable the realization of improvements recommended by the players. Nonetheless, case-by-case analyses suggest caution in using such a diagnostic as a selection tool in talent development programs.

## Introduction

In complex sports like soccer, the identification of talents is often based on coaches' experience and judgements (1). In addition to these subjective assessments, talent identification and development (TID) research aims to provide scientifically sound objective diagnostic tools for assessing potential talent predictors. For example, decision-making skills are considered as relevant talent predictors in elite youth soccer (2) enabling players to select the best action in specific game situations (3). Empirical findings demonstrate that highly skilled youth players possess superior decision-making skills compared to lower-skilled players (4, 5). However, (i) developing as well as (ii) evaluating diagnostic tools for monitoring decision-making skills in TID programs continues to be a major challenge due to general methodological issues (e.g., 6) and insufficient empirical evidence concerning the prognostic validity of cognitive factors (7).

One core challenge for the (i) *development of a decision-making skills diagnostic* tool is the ecological validity addressing the question “[h]ow well a test relates to actual sporting performance and matches the athletes real sporting context” (8, p. 4). Here, both the type of response capturing (e.g., motor vs. verbal responses) and the type of stimulus presentation are relevant (9, 10). The present study focuses on the development of appropriate stimuli for such cognitive skills tests. In this context, the Expert Performance Approach (11) suggests that domain-specific stimuli provide the most reliable and valid diagnostic results (3, 12). In line with that, a meta-analysis by Kalén et al. (7) demonstrated a better suitability of sport-specific stimuli compared to non-specific stimuli for discriminating between higher and lower skilled athletes.

The majority of empirical studies embedded within the Expert Performance Approach presented sport-specific stimuli on 2D video screens. This restricts a realistic presentation of the respective game-situation shown to the players in two ways: First, when filming real decision-making situations to create video stimuli only the environment within the corresponding camera recording angle is captured and, second, when engaged in the experiment participants' head movements do not correspond to a change in the visual field (13). As a result, participants are potentially confronted with less realistic environments, thus limiting the ecological validity of the diagnostic setting (14, 15). This limitation of video screens leads to insufficient opportunities to simulate players' 360° view on the pitch. Yet, Jordet (16) demonstrated that soccer experts use a specific gaze strategy (with a high visual exploration frequency) suggesting that pre-orientation (“scanning”) needs to be considered as an important contributor to soccer players' successful decision-making on the pitch. Subsequent research supported this notion. For example, Phatak and Gruber (17) observed midfielders' behavior and found a positive correlation between pre-orientation and the percentage of successful passes, as well as a negative correlation between pre-orientation and losing the ball. Moreover, it seems that a higher number of head turns before receiving the ball promotes faster decision-making (18) and a higher scanning frequency enhances the probability of pass completion (19).

New technologies such as 360° video footage and head-mounted displays (HMD) enable researchers to design laboratory settings that allow participants an all-around view of simulated soccer situations from a first-person perspective. According to Lindsay et al. (14), this 360° VR technology creates more realistic and immersive test or training environments by “providing visual information that is more representative of competitive experiences” (p. 1). Representative stimuli are supposed to promote ecological validity in terms of transfer to real sport situations [for an overview see Hadlow et al. (20)]. Attributes of representative stimuli are, for example, the presentation of real-world situations from a first-person perspective (21), a high degree of realistic visual information from these situations (22), and different opportunities for action (as in real game-situations) (23). As a further indicator for representative experimental settings used to investigate decision-making skills in sport, Lindsay et al. (14) point out that 360° VR technology potentially supports perception-action coupling processes (24). As such, environmental information presented in the 360° video stimulus regulate head movements (i.e., motor processes) directly influencing the information picked up during the decision-making situation.

Underlining the relevance of representative stimuli, studies using 360° VR tools (as a training tool) without a real motor response demonstrated positive effects for anticipation in cricket (25) and decision-making performance in sport games (26, 27). In addition, an intervention study on decision-making skills of Australian football umpires found stronger engagement from the participants in terms of psychological states such as fidelity, enjoyment, and relevance when using a 360° VR tool (28). Due to a higher level of “presence” in the situation (i.e., psychological experiences of “being there”) and “immersion”, these aspects can be assumed to lead to more valid results compared to presentations on 2D screens (29, 30).

The (ii) *evaluation of a new diagnostic tool* should include players' subjective experiences during the diagnostic process as high level of acceptance and associated motivation increase the likelihood that players may perform at their best (31). Although this is a requirement for generating meaningful data, addressing this objective is not yet common in comparable evaluation studies [for an exception see Krupitzer et al. (32)].

Further issues regarding the evaluation of new diagnostic tools are related to methodological topics of TID research such as psychometric requirements (e.g., satisfactory indicators of reliability), study design features, or different perspectives of statistical analyses. As newly developed diagnostic tools may be used for estimating youth players' current as well as future performance level, such instruments should be evaluated using cross-sectional as well as prospective study designs. When planning evaluation studies, researchers should further consider that TID programs promote youth players on an already advanced performance level, and respective stakeholders aim to receive information to further differentiate these highly selected players. Thus, the evaluation of diagnostic tools used in TID must not be limited to the often-reported comparisons between experts and novices. Rather, a “restriction of the range of talent” (33, p. 13)—achieved via comparisons of experts with at least intermediate youth players—may provide more realistic (although statistically smaller) effect sizes representative of the “real world” of TID (34). In addition to these effect sizes for group-based mean differences, sensitivity analyses can further offer important information to estimate the predictive power of diagnostic tools in TID research (e.g., 35). Considering that decisions in sport practice are often determined on a single-case evaluation of players and that studies investigating high-performing youth athletes are often characterized by small sample sizes, data analysis should also take the single-case perspectives into account when evaluating diagnostic tools (36).

In summary, recent research indicates that soccer-specific 360° video stimuli presented in an HMD have the potential to provide more ecological valid stimuli for diagnostic tools assessing decision-making skills in soccer. However, there is a lack of empirical evidence regarding the psychometric properties (in particular diagnostic and prognostic validity) and more informal criteria such as players' subjective acceptance of the test procedure, influenced by a high degree of presence and appropriate immersion. Thus, the present study aimed to evaluate a newly developed diagnostic tool using an HMD to present players 360° videos as an all-around simulation of soccer-specific situations from a central midfielder's perspective in a TID setting. As elite youth academy (YA) players of professional clubs are the target group for this diagnostic tool, the scientific evaluation was conducted in this high-performance context and addressed three research questions:

1. How do YA players evaluate the tool with the HMD-presented 360° videos concerning immersion and presence?
2. Does the tool demonstrate diagnostic validity in terms of concurrent soccer performance level and age?
3. Does the tool demonstrate prognostic validity regarding future success in adulthood?

*Objective 1* examines the acceptance and added value of the diagnostic setting from the viewpoint of YA players. Players' acceptance supports their motivation to achieve their best possible performance during the diagnostic procedures which is, from the perspective of practice-oriented research, essential for the implementation of a diagnostic tool in the “real world” (37). Concerning the analysis of the diagnostic validity (*Objective 2*), we hypothesize that YA players performing in the highest national league achieve better results in the decision-making skills diagnostic than regional league (RL) youth players, and that more experienced U19 players outperform less experienced U17 players. Moreover, evaluating the prognostic validity (*Objective 3*), we expect that YA players who will play at a higher adult level in the future achieve better diagnostic results than YA players who will play at a lower adult level.

## Methods

### Sample and design

During the 2018/19 season, a total of *N* = 48 male youth soccer players of the U17 and U19 age groups participated in this study. The main sub-sample consisted of *n* = 24 elite YA players competing in the highest national league in their age groups (German Youth Bundesliga). The other sub-sample consisted of *n* = 24 RL players playing in the mid-level league “Bezirksliga” (4th German youth soccer league). The RL players represented the “intermediate” performance level for the evaluation of the diagnostic validity. Both sub-samples were of similar age [*M_YA_* = 17.13 ± 0.76 years vs. *M_RL_* = 17.04 ± 1.30 years; *t*(37.253) = 0.29; *p* = .78] and had a similar age at the entry point into a soccer club [*M_YA_* = 5.95 ± 1.55 years vs. *M_RL_* = 6.00 ± 1.91 years; *t*(41) = 0.10; *p* = .92]. While objective 1 was addressed utilizing only YA players, objective 2 was evaluated within a balanced *cross-sectional 2 × 2 design* (age group x performance level) with each *n* = 12 players from a U19 YA (*M* = 17.67 ± 0.49 years), U17 YA (*M* = 16.55 ± 0.52 years), U19 RL (*M* = 18.08 ± 0.79 years) and U17 RL team (*M* = 16.00 ± 0.74 years).

A *3-year-prospective design* was implemented to evaluate the prognostic validity of the diagnostic tool (objective 3). YA players' future success was operationalized by determining their adult performance level in the 2021/2022 season. Information was found for 22 out of 24 players by searching on “transfermarkt.de”. It is likely that the two players not found either quit their careers or played in such a low league that their status was not documented. Using a median split approach, the variable “achieved adult league” was dichotomized to define the adult performance level “League 1–4” (*n* = 13) and “League 5 or below” (*n* = 11, including the two players not found in the database).

The study was positively evaluated by the first author's university's ethics committee. Participants and their parents provided informed consent for the collection and scientific use of the data. As a precaution, individuals with previous epileptic attacks or cases of epilepsy in the family were excluded from participation.

### Decision-making test

**Figure 1:**
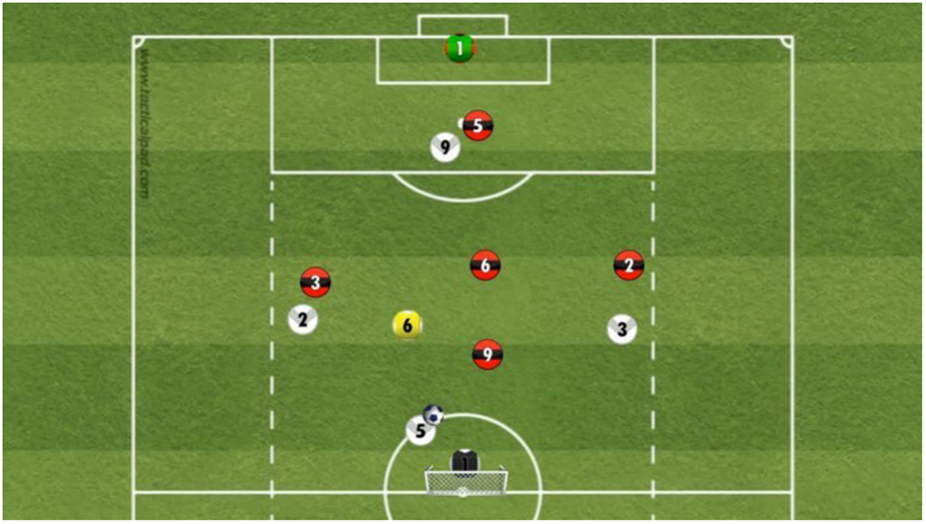
Schematic illustration of a 6 vs. 6 soccer sequence. The central midfield player (No. 6; highlighted in yellow and a member of the “white team”) represents the position of the 360° camera.

To develop the decision-making test, 27 scripted 6 vs. 6 sequences were filmed on one half of a soccer field with a 360° camera (Insta360, Irvine, USA) using instructed players of a U19 German Youth Bundesliga team (see Figure 1). To ensure that the recorded sequences are representative of real game situations, all sequences were created in collaboration with three highly experienced soccer coaches (UEFA Pro-Level License) who also determined the best solution for each decision. Only sequences were used for which an agreement by all coaches was achieved for the best solution for the presented decision task. Compared to a real (competition) match, the reduced 6v6 game situations ensured that the video footage presented the filmed players large enough in the HMD. The camera was placed at the central midfielder's position (No. 6) of the team in ball possession. After the ball was passed a few times within the team, the ball was then played to the camera either by the central defender (No. 5), the left defender (No. 2), or the striker (No. 9). When receiving the ball, five options were available for the central midfielder to continue the play, i.e., to keep the ball or to pass to the right defender, central defender, left defender, or striker.

The game sequences were presented in an HMD (Vive Pro, HTC Corporation, Taoyuan, Taiwan) as omnidirectional 4k video footage on the inside of a sphere that envelops the user's field of view. During the pass to the camera position, the ball was marked with one of three possible colored points for 500 ms. This was done based on experiences from pilot studies to avoid unnatural gaze behavior (i.e., looking around without observing the ball). After the ball reached the camera, the video terminated, and the image turned black. Each video lasted about 10 s.

After detailed instructions and four practice trials for familiarization, participants were asked to complete 54 test trials in two blocks (27 videos x 2 presentations). The presentation order of the video clips was pre-defined and while it differed between the blocks, it was kept identical for all participants. After each video termination, participants were required to respond verbally by stating the color mark on the ball and how they would best continue playing. Only if both statements were correct, the answers were also counted as “correct”. The percentage of correct decisions across the 54 trials was determined as the performance outcome (decision accuracy in %). A split-half reliability estimation revealed acceptable internal consistency for the assessment of this performance outcome (*r *= .78; split-half method, Spearman-Brown corrected). The decision-making test lasted about 45 min.

### Participants' subjective evaluation of the setting

Quantitative evaluations and qualitative interview data were gathered to receive YA players' feedback on their experience with the newly developed diagnostic tool. For this purpose, participants completed a 5-point Likert scale questionnaire (see Table 1). The questions were adopted from studies assessing immersion and presence in VR environments (38, 39).

**Table 1:** Mean values and standard deviations of the YA players’ (*n* = 24) subjective evaluations [agreements ranging from 1 = “not at all” to 5 = “totally”; modified according to Chertoff et al. (38); Witmer et al. (39)].

Item	Mean value ± SD
How exciting was the task?	4.42 ± 0.79
How involved did you feel in the game situation?	4.08 ± 0.69
How much did you feel like being part of the game?	4.04 ± 0.69
To complete the task, how much did you have to engage with video environment?	3.77 ± 1.25
How fast did the time pass?	3.83 ± 0.82
How much did you forget about the physical reality around you?	3.54 ± 1.05

Additionally, qualitative interviews were conducted to gather further information on the strengths and weaknesses of the setting. The YA players were asked (a) how they liked the setting, (b) whether they could imagine integrating VR-based training into their everyday life, and if so, in what form. Lastly, the players were asked (c) what they thought could be done to improve the setting. The questionnaire took approximately 5 min to complete, the subsequent interviews lasted about 10 min.

### Statistical analysis

The *quantitative data* was analyzed using SPSS 28 (IBM, 2021). To examine the criterion validity (i.e., objectives 2 and 3), one-tailed alpha levels for hypotheses testing were set at .05. Cohen's *d* and *η*^2^ served as effect sizes.

T-tests for paired and independent samples were performed to compare YA players' ratings between the items in the quantitative evaluation questionnaire (objective 1) and the adult performance levels regarding the performance in the diagnostic (objective 3). Considering the small sample sizes for these comparisons, inferential decisions were further secured by additional non-parametric testing (that led in all cases to the same statistical conclusion). A two-way analysis of variance (ANOVA) was conducted to examine the diagnostic validity (objective 2) within the 2 × 2 design with the factors performance level (YA vs. RL) and age group (U19 vs. U17).

As small sample sizes are often found in expertise research (40), the statistical power for testing the validation hypotheses was determined. *Post hoc* power analyses (G*Power version 3.1.9.7.; 41) revealed an acceptable statistical power for objective 2 (*1-β* = 86%) and low statistical power for objective 3 (*1-β* = 60%) even if large effect sizes (*d *= .80) for the differences between each of the two considered groups were assumed in the investigated population. In addition to the inferential group-level analyses, descriptive single-case results were presented for a more detailed insight into the results.

A receiver operating characteristic (ROC) curve was then utilized to illustrate the prognostic power in relation to all possible values of the true positive rate (sensitivity) and the false positive rate (1-specificity) (42). To estimate the quantitative accuracy of the diagnostic tool, the “area under the ROC curve” (AUC) was determined and tested for a significant deviation from the chance diagonal [i.e., the 45° line through the coordinates (0, 0) and (1, 1) covering 50% of the area]. For each point of the ROC curve, the Euclidean distance to the optimal expression of sensitivity and specificity was calculated to detect the ideal cut-off value, i.e., the point with the smallest distance that discriminates players regarding their adult performance level. This cut-off value was used to calculate the odds ratio (*OR*) that quantifies the strength of the relationship between the performance in the diagnostic and later adult success.

Finally, the *qualitative data* was analyzed utilizing a summative content analysis to describe and quantify players' statements (43). For this purpose, the interview schedule was used as a basic coding scheme (categories of “immersion” and “presence”).

## Results

### Objective 1: participants' subjective evaluation of the setting

Overall, YA players achieved a decision accuracy of *M *= 69.06% (±9.08%) which is indicative of their general understanding of the 360° video simulations and the experimental test procedure. Aligning with these results, the diagnostic setting was positively evaluated by the 24 YA players with an average sum score of *M *= 3.95 (±0.54) across all items. Especially the first three questions were rated highly (“How exciting was the task?”, “How involved did you feel in the game situation?”, “How much did you feel like being part of the game?”; see Table 1). The question “How exciting was the task?” was rated significantly higher than all other questions [2.49 ≤ *t*(23) ≤ 3.08; .005 ≤ *p* ≤ .021.; 0.70 ≤ *d* ≤ 1.39] with the exception of the second item pertaining to the involvement into the game situation. However, the latter was still significantly higher rated than question five “How much did you forget about the physical reality around you?” [*t*(23) = 2.69; *p *< .05; *d* = 0.99].

Additionally, the analysis of the qualitative data suggested an overall consensus that completing the decision-making tasks presented in HMD was perceived as highly enjoyable by the YA players. Players pointed out that working with the diagnostic tool offered a change from everyday training, that they easily got immersed in the game situation, and that the pre-orientation task was easy to perform. While it should not take up too much time, all players stated that they would appreciate practicing and training their decision-making skills with an HMD. Often, one session per week with a maximum of 15 min was recommended. Regarding the appropriate timing of such additional training in their daily routine, players mentioned the time before training (*n *= 8), after training (*n *= 6), or before going to bed (*n *= 3). Some players could imagine using the tool during training or before a match, others only on the off day.

Only a few players mentioned suggestions for improvement. Some players expressed the desire for more game situations including the perspectives of other playing positions (*n *= 4). Further feedback was related to the improvement of the video quality (*n *= 4), and the availability of ambient sounds (*n *= 2). Few players would have liked to be able to move in the video environment or play a pass like in a real soccer match (*n *= 2). The limited field of view inside the HMD (≈110°) was hardly criticized (*n *= 1).

### Objective 2: diagnostic validity

Table 2 presents the descriptive results regarding the concurrent diagnostic validity. YA players (69.06% ± 9.08%) performed better than the RL players (58.80% ± 8.55%), and U19 players (67.13% ± 8.55%) had a higher decision-making accuracy compared to U17 players (60.73% ± 10.77%). The 2 × 2-ANOVA confirmed the underlying hypotheses and revealed significant main effects for performance level [*F*(1, 44) = 18.07, *p *< .001, *η*^2^ = .29] and age group [*F*(1, 44) = 7.04, *p *< .01, *η*^2^ = .14]. The larger performance level effect size is accompanied by superior diagnostic results of the U17 YA compared to the U19 RL players (see Table 2). The performance level x age group interaction was not significant [*F*(1, 44) = 0.12, *p = *.73].

**Table 2:** ANOVA results regarding diagnostic validity (left side) and descriptive results for decision-making accuracy (%) separated for the four teams (right side).

	Age group			Performance level			Interaction				YA		RL	
	*F*(1,44)	*p (one-tailed)*	*η* ^2^	*F*(1,44)	*p (one-tailed)*	*η* ^2^	*F*(1,44)	*p*	*η* ^2^		U19	U17	U19	U17
Accuracy	7.04	<.01	.14	18.07	<.001	.29	0.12	.73	.003	M	72.69	65.43	61.57	56.02
										SD	6.52	10.06	6.57	9.64

**Figure 2:**
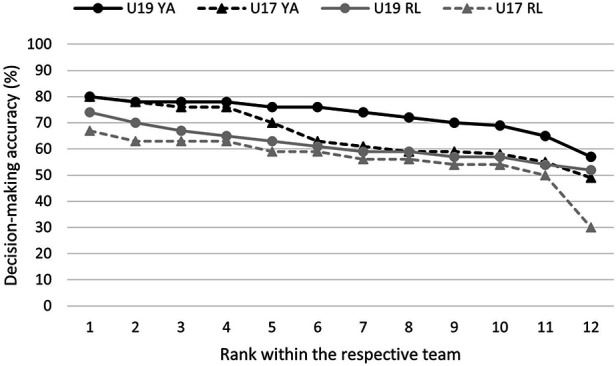
Decision-making accuracy (in %) for each player separated for the four teams (*n* = 4 × 12; players are ordered by their ranked performance in the diagnostic within their team).

Figure 2 illustrates the ranked single performances in the diagnostic separately for each of the four teams. This detailed single-case perspective confirmed that the U19 YA players performed better and the U17 RL performed worse than the other groups. In line with the hypotheses, the ranked order of the U19 RL players' performance was higher than that of the U17 RL players. The U17 YA players are the only group that showed a division in its performance ranking: Whereas the top four U17 YA players performed on the highest level of the whole study sample (i.e., similar to the four best U19 YA players), the players ranking at 6 or worse in the U17 YA team performed on the same level as the corresponding U19 RL players.

### Objective 3: prognostic validity

**Figure 3:**
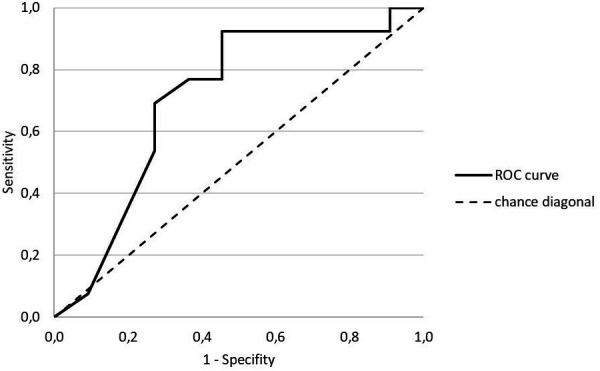
Receiver operating characteristic (ROC) curve for the prognostic validity of the YA players’ decision-making results.

The prospective analyses demonstrated a significant difference in the decision accuracy between the 13 in adulthood more “successful” and the 11 less “successful” YA players [*M_League 1–4_* = 72.22% ± 7.52% vs. *M_League 5 or below_* = 65.32% ± 9.66%; *t*(22) = −1.97; *p* < .05; *d* = 0.80]. The ROC curve resulted in an *AUC* = 0.71 indicating a 71% chance for a correct assignment of the YA players to the two adult performance levels based on the diagnostic results (Figure 3). The one-sided tested AUC value was significantly different from the chance diagonal [*p <* .05; *LL CI* (90%) = .52]. An optimal cut-point value of 0.71 was determined and resulted in *OR* = 6.00. Thus, YA players with 71% or more correct decisions had a six-times higher chance of reaching “League 1–4” in adulthood compared to players with fewer correct decisions.

**Figure 4:**
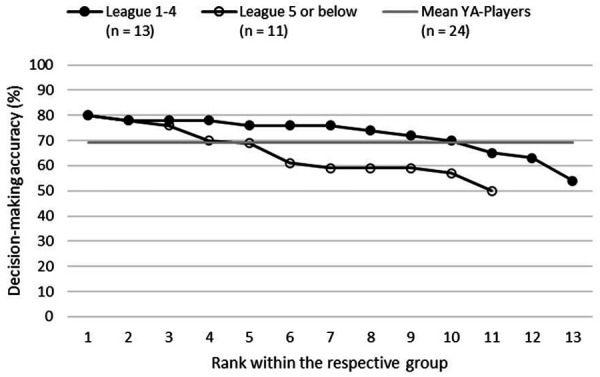
Decision-making accuracy (in %) for each YA player separated for the achieved two adult performance levels (the players are ordered by their ranked performance in the diagnostic within their adult performance group) and the mean performance of all YA players.

Figure 4 illustrates the ranked single performances in the diagnostic test separately for both adult performance levels. Again, this single-case perspective confirmed the initial assumptions concerning the group-mean-based difference. Yet, it also provided further information concerning the best performers in the diagnostic: The top three players of the lower adult performance level achieved as excellent diagnostic results as the top players of the higher adult performance level. Only from rank 4 onwards, the differences become larger in favor of the more “successful” players and, for example, the “League 1–4” player placed on rank 10 in his group performed equally well as the “League 5 or below” player ranked on 4. Concerning the overall mean performance of the YA players (69.06% ± 9.08%), already the “League 5 or below” player ranked on 5 in his group performed below average, while this was only the case for the “League 1–4” player in 11th place.

## Discussion

The present study evaluated a diagnostic tool assessing decision-making skills in soccer using HMD technology to present players 360° videos of soccer-specific situations from a central midfielder's perspective. Although information about reliability is vital for newly developed diagnostic tools (44), it is often not reported in this field of research (20). The assessment of the diagnostic tool's performance outcome demonstrated an acceptable split-half reliability (*r* = .78). This result is slightly above the reliability of a decision-making test in which players had to respond with a motor action (dribbling the ball and playing a pass) to video stimuli (5). Grounding on this psychometric prerequisite, elite YA players' subjective experience with the diagnostic tool (*objective 1*) and statistical indicators for the criterion validity (*objectives 2 and 3*) were evaluated.

### Youth academy players' subjective evaluation of the setting (objective 1)

Participants' subjective evaluation of an experimental setting has rarely been explored in previous research. Thus, the present study addressed a call by Richlan et al. (45) who urged for assessing players' perceived immersion and motivation in order to gather information potentially explaining the achieved effects within a study. Players' positive evaluation of the new diagnostic setting is imperative for the successful implementation of such diagnostic (or training) tools in the long term. On average over all items, the quantitative evaluation demonstrated positive ratings by the 24 YA players (*M *= 3.95 ± 0.54). Ratings of the single items indicated that the diagnostic setting generated a high motivational effect and involved the players in the game situations. Additionally, the qualitative evaluation underlines YA players' acceptance of the newly developed diagnostic tool and revealed players' high level of enjoyment. As a result, all interviewed players could imagine a decision-making training with the 360° VR technology.

The higher motivation stated by players in the setting is likely generated by the innovative character of the HMD and 360° videos. Focusing on the cognitive demands without having an additional physical load may have also contributed to the acceptance due to the high training volumes at youth academies. Motivating diagnostic (or training) tools that do not require any physical load may also be an opportunity for currently injured players who are not able to participate in training on the field. This may contribute to the convalescents' well-being as injured YA players are provided with soccer-specific training opportunities even in this phase. This may be of particular importance since injured athletes often feel insufficiently addressed by the coach during the injury period (46).

Moving forward, possible improvements mentioned by the YA players were primarily related to technical aspects and should be considered for further development of the diagnostic setting. Some improvements seem easy to realize. For example, ambient sound can be implemented while the video is recorded and may provide intriguing future research perspectives (e.g., examining the role of auditive support from teammates). A further increased video quality will become possible resulting from ongoing technological advancements. The preferences of a few players for improved video quality may be attributed to the vergence-accommodation-conflict (47) which might have led to a distorted perception of depth in a video image, especially in an HMD where the image is close to the eyes. The consequence is a blurred image of the entire virtual environment (48). To address this problem, the industry is developing gliding displays for HMDs to enable a more natural focus on virtual objects at any distance.

Other suggested improvements seem technologically more difficult. The desired feature of capturing a motor response that represents a real-world pass is feasible by using a foot tracker (e.g., HTC Vive Tracker). Yet, coupling the foot tracking data with the presentation of the 360° video remains challenging. Moreover, participants' unrestricted freedom of movement seems hardly possible to implement into the experimental setting as players still need to wear the HMD.

### Diagnostic and prognostic validity (objective 2 and 3)

The cross-sectional results confirmed the two hypotheses regarding the *diagnostic validity*: YA players performed better than RL players and more experienced players (U19) outperformed less experienced players (U17). Additionally, it was found that YA players of both age groups achieved better test results than the RL players. Consequently, the performance level effect (*η*^2^ = .29) was larger than the age effect (*η*^2^ = .14). This may indicate that the diagnostic tool addresses soccer-specific components to a greater extent than age-related components what would be in favor of the diagnostic tool insofar as age-related increases might be caused by natural maturation and not by soccer-specific experiences [e.g., for executive functions, see Beavan et al. (49)]. Moreover, the effect size of the performance level effect (*η*^2^ = .29 converted to Hedges' *g* = 1.28) was distinctly higher than the effect size for differences in decision-making outcomes between higher and lower-skilled players reported in the meta-analysis by Kalén et al. (7) (Hedges' *g* = 0.84 for cross-sectional studies using sport-specific stimuli).

The prospective results of this study also confirmed the hypothesis regarding the *prognostic validity*: Thus, YA players who play 3 years later at a higher adult level (“League 1–4”) achieved better diagnostic results than YA players who play at a lower adult level (“League 5 or below”). The detected effect size (*d* = 0.80) should be noticed even more as a high-level and thus homogeneous sample of elite YA players (top 1% in Germany) was investigated over a mid-term prognostic period (50). Looking at the existing knowledge about the prognostic relevance of diagnostic tools in this field, this is an important indicator for the promising potential of the new cognitive performance diagnostic: Kalén et al. (7) identified in their meta-analysis only three existing prospective studies, and these found either no significant differences between sub-elite and elite players (51) or only small to medium effect sizes (5, 52).

As for the further statistical perspectives, two additional analyses complemented the “traditional” group-based analysis of criterion validity in this study. First, *single-case considerations* provide deeper insights explaining differences in group means. Regarding the cross-sectional results (objective 2), the on average better performances of the U17 YA players compared to the two RL teams are mainly based on the test results of the best five players. Here, the top 4 players of the U17 YA performed on the level of the older top 4 players of the U19 YA. Regarding the prospective analysis (objective 3), the illustration of single-case performances revealed that the top 3 players of both future performance level achieved similar diagnostic results. Only from rank 4 onwards, the later “successful” players outperformed the “less successful” players.

Second, the relevance of the prognostic validity was underlined by a ROC curve and a calculation of the AUC. This *sensitivity analysis* indicated a probability of 71% of correct assignments to the future performance level. Moreover, YA players with good diagnostic results (in relation to an optimal cut-off value) had a six-times higher chance to play in one of the first four leagues in adulthood. ROC curve and AUC calculations have rarely been reported in sports science as indicators for sensitivity (for exceptions see e.g., 35, 53). However, such information about sensitivity is particularly useful in the early stages of the development of a new diagnostic tool (42) to determine an appropriate cut-off, affecting the sensitivity and specificity of the test (54). However, for TID research it should be noted that in terms of sensitivity and specificity, equal weighting of “false positive” and “false negative” classification errors is not always appropriate. Thus, defining optimal cut-off values should be reconsidered for each application as decision errors may have specific negative consequences (34).

Taking the results of this study together, the new decision-making skills diagnostic tool provides an added value to former tools using 2D screens by expanding the variety of soccer-specific situations which could be simulated (e.g., typical decision-making tasks for central midfielders that receive the ball from a defender and should be aware of their surrounding environment). Underlining the representativeness of the stimuli, players' subjective evaluation indicated high acceptance of and immersion in the setting. By creating more representative video stimuli, the present study addressed only one, albeit a very important, demand for more ecologically valid experimental settings (7, 9, 10). Regarding the second demand (i.e., representative response capturing), this study followed recommendations by Kredel et al. (9) who encouraged practicable compromises balancing high degrees of ecological validity and experimental control. Although capturing players' decision-making by a verbal response and not through a real-world motor action, the detected effect sizes regarding criterion validity and sensitivity analyses as well as the single-case considerations provide empirical evidence for the diagnostic. Against this background, the effect sizes may be interpreted as conservative estimators for the superior performance of (older and in the future more successful) YA players in decision-making skills. Moreover, in addition to former studies (25–28) the results provide further evidence supporting the notion that perception-action coupling through motor responses is not necessary for sufficiently valid sport-specific 360° VR tools. Thus, perception-action coupling processes may already be initiated by the exploration of the HDM presented 360° environment with head movements (14) or by action planning processes as the motor system is active before an action is actually carried out (12).

Perspectively, the development of 360° VR training tools for elite youth soccer seems promising but also still challenging. In other domains, such as surgery (55) or rehabilitation (56), the use of VR and HMD for skills training is an already established method. However, although there is preliminary evidence that athletic skills learned in virtual environments can be transferred into sport practice (57, 58), it remains unclear whether complex sensorimotor skills can be effectively trained using this technology [for an overview see Richlan et al. (45)]. This is further complicated by the fact that generalizable statements on the effectiveness of training programs are hardly possible due to the different skills that are required in different sports. Rather, it is necessary to validate each sport-specific training program separately (59). Thus, in the context of designing a cognitive training program, the most urgent question to be answered is, to what extent a transfer of training effects generated in the virtual environment to the real world on the soccer field is possible (59–61).

For future developments of 360° VR diagnostic and training tools, technological advancements regarding the field of view presented in the HMD, the additional recording of participants' eye movements and the use of computer-generated imagery for the stimuli creation may help to overcome some limitations of the present study caused by the technological standard existing at the time of data assessment for this study.

### Technological perspectives

HMD projections of 360° videos still have limitations regarding the restricted field of view (FOV). While healthy people's horizontal FOV is about 180° (62), the HTC Vive Pro projection limited this to about 110° in this study. This aspect is an important issue in the discussion about research using HMD (e.g., 63). Yet, it was only criticized by one YA player in the qualitative interviews indicating that—despite the limited FOV—the diagnostic setting provided a feeling of being “involved” in the 360° environment. For future studies, limited FOV will probably not be an important issue anymore as HMD devices with a larger horizontal FOV up to 210° have been developed meanwhile (e.g., StarVR One, StarVR Corporation). Moreover, studies may also take the limited FOV as an experimental factor (i.e., varying the FOV to explore the use of shoulder glances or peripheral vision) or training factor (i.e., intended limitation of FOV to challenge players more to use head movements or shoulder glances for pre-orientation).

Concerning the analysis level of this study, the evaluation focused on decision-making accuracy on the behavioral outcome level without addressing the underlying cognitive process level. HDM technologies with integrated eye-tracking systems enable the additional assessment of eye and gaze movements. Eye-tracking provides highly dimensional process data that can be analyzed by more complex statistical methods to gain new insights regarding eye movement-based expertise recognition. For example, in a study investigating soccer goalkeepers' decision-making skills in typical build-up situations, it was possible to assign the goalkeepers to the expert, intermediate, and novice performance levels with an accuracy of 78.20% based on eye-tracking data analyzed with machine learning algorithms (64). In addition, a deep learning approach explored latent perceptual features in fixation image patches of goalkeepers and identified expertise level with 73.11% accuracy (65).

Computer-generated imagery (CGI) techniques may provide an alternative to 360° videos for simulating decision-making tasks in HMD in the future and there are already tools available using these techniques (e.g., Be Your Best). Options for importing positional tracking data of real soccer matches as well as the potential to implement motor responses by motion tracking systems in the experimental setting are promising perspectives offered by VR tools using CGI. The (compared to 360° video stimuli) technologically easier coupling of participants' motion tracking may enable interaction processes between the whole-body movements of the user and their CGI generated virtual environment. This may simulate perception-action coupling processes that are even more representative than potential perception-action coupling processes induced by head movements in this study. However, CGIs are so far still challenged to reproduce real biological movements for the players presented in the animation as human perception is extremely sensitive to the detection of these movements (66). Nevertheless, future technological progress will probably eliminate this limitation someday, and new VR applications will emerge which facilitate the creation of specific situations for different playing positions such as defender or striker (57).

## Conclusion

This study demonstrated empirical evidence for a diagnostic tool using soccer-specific 360° videos presented in an HDM. Going beyond former studies using 2D screen projections, this new diagnostic tool provided participants with a game-like “all-around view” from the viewing perspective of a central midfield player. YA players' subjective feedback demonstrated players' general acceptance of the use of such a tool in an elite youth academy. Based upon sufficient psychometric reliability (*r* = .78), the study detected a performance level effect between elite and intermediate youth players (*η*^2^ = .29) and an age effect between U19 and U17 players (*η*^2^ = .14). Moreover, a noticeable effect size (*d* = 0.80) was found in the diagnostic results within elite YA players (top 1% in Germany) discriminating players that achieve different performance levels in adulthood 3 years post the assessment. Compared to former studies, the large effect sizes for the diagnostic and prognostic validity underline the assumption that (expertise) effects increase with more representative stimuli. Thus, the applied technology promises added value to 2D video screen projections for practitioners' and sport scientists' examination of youth players' decision-making skills.

From an applied perspective, the technology provides opportunities to investigate more soccer-specific situations demanding an all-around view. Thus, important aspects for good decision-making in soccer such as the “pre-orientation” (e.g., by glancing over shoulders) could also be considered in laboratory settings. Furthermore, future technological advancements will enable the realization of improvements recommended by the players as well as the assessment of underlying cognitive processes (eye-tracking) or more comprehensive perception-action coupling processes within VR stimuli generated by CGI.

From a TID research perspective, these results should not be overinterpreted as a foundation for a “selection tool” in talent identification. The single-case analyses presented in this study demonstrated that some players with good diagnostic results were not successful 3 years later, whereas some other players with worse results did achieve high adult performance level. Because of these always existing “false positive” and “false negative” diagnostic results, caution is suggested in using such a diagnostic as a selection tool in TID programs (6, 67). Rather, the main purpose of these kinds of diagnostic tools should be seen in the monitoring of prognostically relevant features, i.e., as a tool supporting the talent development process.

## Data Availability

The raw data supporting the conclusions of this article will be made available by the authors, without undue reservation.
